# Intratendinous Injection of Autologous Conditioned Serum for Treatment of Lateral Epicondylitis of the Elbow: A Pilot Study

**DOI:** 10.34172/aim.2022.52

**Published:** 2022-05-01

**Authors:** Deniz Ipek, Murat Çalbıyık, Sinan Zehir

**Affiliations:** ^1^Department of Orthopaedics and Traumatology, Faculty of Medicine, Hitit University, Çorum, Turkey

**Keywords:** Autologous Conditioned Serum, Elbow, Lateral Epicondylitis, Orthokine

## Abstract

**Background:**

Autologous conditioned serum (ACS) has been effectively used in treatment of osteoarthritis. However, less is known about its efficacy in tendon disorders. In this pilot study, we aimed to evaluate the short- and long-term effects of intratendinous injection of ACS in lateral epicondylitis (LE) of the elbow.

**Methods:**

This prospective cohort included 42 patients with LE of the elbow who received 4 intratendinous injections of Orthokine^®^ (Orthogen Lab Services GmbH, Düsseldorf, Germany) under local anesthesia over 2 weeks in an outpatient setting. The clinical and functional outcomes of injections were evaluated at 3 months and 1 year after the procedure. Pain was assessed using a visual analog scale (VAS) and functional assessment was made using the Mayo Elbow Performance Score (MEPS) and Oxford Elbow Score (OES).

**Results:**

The pre-injection VAS score (7.07±1.19) improved significantly after the procedure at both 3 months (3.55±0.56, *P*<0.001) and 1 year (1.73±0.82, *P*<0.001). Similarly, the mean MEPSs were significantly different between baseline and 3 months (56.42±7.51 vs. 79.76±3.81, *P*<0.001) and between baseline and 1 year (56.42±7.51 vs. 94.28±4.06, *P*<0.001). The baseline OESs (84.17±6.07) also improved with intratendinous injection of ACS at 3 months (41.96±9.23, *P*<0.001) and 1 year (7.43±4.31, *P*<0.001). Only six patients (14.2%) had mild ecchymosis and swelling around the injection site which resolved spontaneously.

**Conclusion:**

ACS is a promising option for treatment of LE of the elbow, given its early onset of pain-relieving action and longlasting functional effects. These findings await confirmation by large-scale and prospective trials.

## Introduction

 Lateral epicondylitis (LE) of the elbow is the most common wrist overuse syndrome in the general population. The condition results from constant strain of the extensor tendons of the forearm, especially of the extensor carpi radialis brevis (ECRB) tendon near its attachment to the lateral epicondyle.^1^ Chronic LE, commonly referred to as “tennis elbow”, is common in tennis players, athletes, office workers and persons who have to keep performing repetitive motions of the wrist such as carpenters, plumbers, painters, and people who use hammers or screwdrivers.^2^ Although the overall incidence is reported to range from 2.4 to 4 per 1000 people, the true incidence is likely much higher since most people with LE do not seek treatment until their symptoms progress.^1^

 The term “epicondylitis” has been suggested to be a misnomer since microscopic evaluation of the tendons of patients with LE demonstrates angiofibroblastic degeneration and collagen disarray, but not inflammation.^3,4^ The actual pathology in LE is hypoxic degeneration of the tendon of the ECRB near its attachment to the lateral epicondyle, which results in inadequacy of the repair mechanisms against chronic trauma.^3,4^ Although there are various non-surgical therapeutic approaches for LE management, with increasing understanding of its pathology, recent therapies have targeted improving the regenerative capacity of the diseased tendon tissue rather than suppressing the presumed inflammation, particularly for the treatment-resistant chronic LE.^3,4^ Among these, autologous proliferative therapies have recently become popular and been suggested as an alternative therapy to improve tendon regeneration in LE of the elbow.^5-7^

 Autologous conditioned serum (ACS), which is currently marketed under the name Orthokine^®^ (Orthogen Lab Services GmbH, Düsseldorf, Germany), was developed to provide a novel injectable solution enriched in Interleukin-1 receptor antagonist (IL-1ra), a competitive antagonist of interleukin-1 (IL-1), which is responsible for several degenerative and inflammatory cascades in chronic joint and muscle disorders, and growth factors.^8^ Several studies have demonstrated its safety and efficacy in treatment of knee and hip osteoarthrosis, but less is known about its effect on tendon disorders.^9,10^ Previous studies on the effectiveness of intratendinous injections of autologous blood and platelet-rich plasma reported conflicting outcomes, some citing significant clinical relief and others reporting no beneficial effect in short-term. Therefore, there is still need for further studies to determine the short- and long-term effects of intratendinous injection of ACS in LE of the elbow.^11-13^

 On this basis, this pilot study aimed to determine the potential role of intratendinous injectionof Orthokine^®^ in treatment of LE of the elbow.

## Materials and Methods

###  Study Design and Patients

 This was a prospective non-comparative pilot study on a cohort of 42 patients (male:female ratio, 21/21; mean age 38.0 ± 6.4 years), presenting with chronic LE to the Orthopedics and Traumatology Department of Hitit University Faculty of Medicine, Corum, Turkey between April 2013 and November 2013. The study was conducted in accordance with the principles set forth in the Helsinki Declaration 2008. The inclusion criteria were age 25–65 years and LE symptoms lasting for at least 6 months despite the use of various conservative treatment options, including non-steroid anti-inflammatory drugs, intra-articular injection of steroids, arm bracings, and extra-corporeal shock wave therapy. Patients with systemic disorders such as rheumatoid arthritis or diabetes, previous history of arthritis or fracture of the elbow, patients who received surgery for elbow tendinosis, and those who received intra-articular injection of steroids within the last 8 weeks or physical therapy within the last 4 weeks were excluded from the study. All patients received intratendinous injection of ACS.

 The effect size was calculated as 0.1793103. According to power analysis, the minimum sample size was calculated as 10 patients with 95% sample power and 0.05 alpha error on G*Power 3.1 for Windows.

###  Treatment Protocol

 Treatment consisted of 4 injections of ACS into the ECRB tendon administered twice a week for 2 weeks. All procedures were performed in an outpatient setting under local anesthesia. The ACS was prepared using the Orthokine^®^ serum preparation kit according to the manufacturer’s instructions (Orthogen Lab Services GmbH, Düsseldorf, Germany). Four 10mL aseptic venous blood samples were drawn from each patient using 4 separate EOT^®^ II syringes. The blood-containing syringes were incubated at 37°C for 6 hours and then centrifuged at 5000 rpm for 10 minutes. The supernatant (Orthokine^®^ serum) was drawn into 4 separate syringes in aseptic conditions and stored at -20°C until injection time. Since all of the material injected during the procedure is derived from the patients’ own blood without adding any foreign substances, no dosage information is provided.

 The injections were performed while the patient was sitting in a comfortable position with the affected arms flexed in front of the chest. The skin of the injection site was prepped and draped. For local anesthesia, 10 mL of 0.5% bupivacaine was injected over the injection area, then 2 mL of the prepared Orthokine^®^ ACS was injected into ECRB tendon using a peppering technique in which the serum was distributed in a clockwise direction into the tendon and the surrounding area. The injection site was closed, and the patient was discharged.

###  Outcome Measures and Follow-up

 Clinical assessment was made just before the procedure and repeated at 3 months and 1 year after the procedure. Patients were asked to evaluate their overall pain perception in the affected elbow using a 10-point visual analog scale (VAS). Functional assessments were made using the Mayo Elbow Performance Score (MEPS) and Oxford Elbow Score (OES). MEPS is a simple 4-item scale that mainly focuses on pain intensity, range of motion, elbow stability, and ability to perform some daily activities using the elbow joint.^14^ OES provides more detailed information regarding functional abilities and pain intensity, and assesses the psychosocial consequences arising from chronic elbow pain and motion restriction.^15^

###  Statistical Analysis

 Scale parameters were described with means, standard deviations, range, and minimum-maximum values. The Kolmogorov-Smirnov Z test with normal probability plot was used for normality distribution of scale data. The Wilcoxon signed-rank test was used for differences between two measurement points for non-normally distributed parameters. The paired samples *t* test was used for differences between two measurement points for normally distributed parameters. All statistical analyses were performed using the Statistical Package for the Social Sciences software for Windows SPSS (SPSS version 16.0, IBM Corporation, Chicago, IL, USA) at 95% confidence interval.

## Results

 The intratendinous injection procedure was well tolerated in all of the patients. Only six patients (14.2%) had mild ecchymosis and swelling around the injection site which resolved spontaneously. None of the patients discontinued the 2-week treatment duration of protocol. Mean age was 38.00 ± 6.40 with 31–56 range. The mean follow-up was 21.2 ± 7.7 months (Table 1).

**Table 1:** Age, Follow-up, VAS, MEPS and OES Parameters of Patients

**Parameter**	**Mean±SD**	**Range (Min-Max)**
Age	38.00 ± 6.40	25.00 (31.00–56.00)
Follow up	21.24 ± 7.68	26.00 (12.00–38.00)
VAS Score		
Preoperation^a^	7.13 ± 0.61	3.00 (7.00–10.00)
3^rd^ month^a^	3.52 ± 0.54	1.50 (3.00–4.50)
1^st^ year^a^	1.71 ± 0.95	3.00 (1.00–4.00)
MEPS Score		
Preoperation^b^	56.43 ± 7.51	30.00 (40.00–70.00)
3^rd^ month^a^	79.76 ± 3.82	10.00 (75.00–85.00)
1^st^ year^a^	94.29 ± 4.07	10.00 (90.00–100.00)
OES Score		
Preoperation^b^	84.17 ± 6.07	22.92 (72.91–95.83)
3^rd^ month^a^	41.96 ± 9.23	62.50 (31.25–93.75)
1^st^ year^b^	7.44 ± 4.32	16.66 (0.00–16.66)

VAS, Visual analog scale; MEPS, Mayo Elbow Performance Score; OES, Oxford Elbow Score. ^a^Non-normally distributed. ^b^Normally distributed (Kolmogorov-Smirnov Z test).

**Figure 1:**
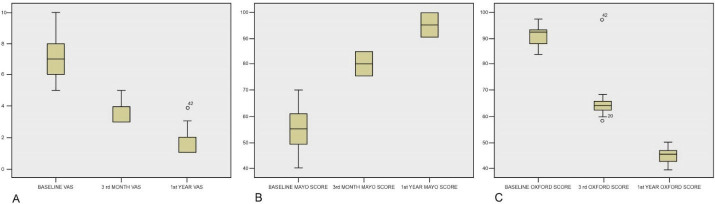
Box-and-Whisker Plot Showing the Changes in VAS Scores (A), MEPS Scores (B), and OES Scores (C) with Intratendinous Injection of ACS Over One-Year. The horizontal line within the box indicates the median, boundaries of the box indicate the 25th and 75th percentile, and the whiskers indicate the highest and lowest values of the results. The mild outliers are marked with open circles (o) and extreme outliers with asterisks (*).

 The mean VAS score, MEPS score, and OES score demonstrated statistically significant improvement at the third month and first year after the intratendinous injection of ACS compared to pre-injection scores (*P* < 0.001). The mean VAS scores were significantly different between baseline and third month (mean difference: 3.60, *P* < 0.001), between baseline and 1st year (mean difference: 5.41, *P* < 0.001), and between third month and first year (mean difference: 1.81, *P* < 0.001). The mean MEPS scores were significantly different between baseline and third month (mean difference: -23.33, *P* < 0.001), between baseline and first year (mean difference: -37.86, *P* < 0.001), and between third month and 1st year (mean difference: -14.52, *P* < 0.001). The mean OES scores were significantly different between baseline and third month (Mean Difference: 42.21, *P* < 0.001), between baseline and first year (mean difference: 76.73, *P* < 0.001), and between 3rd month and 1st year (mean difference: 34.52, *P* < 0.001) (Table 2 and Figure 1).

**Table 2:** Preoperation, 3rd Month and 1st Year Differences of VAS, MAYO and OES Scores (*P* Values)

**Difference**	**VAS Score**		**MEPS Score**		**OES Score**	
	**Mean Difference**	***P***	**Mean Difference**	***P***	**Mean Difference**	***P***
Preoperation-3^rd^ month	3.60	< 0.001^a^	-23.33	< 0.001^a^	42.21	< 0.001^a^
Preoperation-1^st^ year	5.41	< 0.001^a^	-37.86	< 0.001^a^	76.73	< 0.001^b^
3^rd^ month-1^st^ year	1.81	< 0.001^a^	-14.52	< 0.001^a^	34.52	< 0.001^a^

VAS, Visual analog scale; MEPS, Mayo Elbow Performance Score; OES, Oxford Elbow Score. ^a^Wilcoxon signed-rank test. ^b^Paired samples t test.

## Discussion

 In this prospective cohort study, we primarily showed that intratendinous injection of ACS was associated with a significant improvement in pain and functional status in both short- and long-term in patients with LE. The treatment was well tolerated by the patients, showing its potential safety and applicability in an outpatient setting. Completing the treatment within 2 weeks and requiring only 4 visits in total helped to achieve good patient compliance.

 Although various surgical and non-surgical treatment methods have been used in clinical management of LE, there is no widely accepted and standardized treatment for LE. Traditionally, surgery is indicated in patients with symptoms persisting for over 6 months.^16^ However, patients with persisting symptoms are less likely to be referred for surgery, given that only 0.7% to 1.5% of patients with chronic LE proceed to surgery.^1^ Since the condition is mainly related to decreased regenerative capacity of the damaged tendon tissue, injection therapies have recently become more widely used. There have been several studies and a number of randomized trials to assess the role of various injection agents, including steroid, platelet-rich plasma, and autologous whole blood, in pain relief and functional improvement in patients with chronic LE.^5-7,12^ However, none of these trials showed enough efficacy to warrant the use of these agents to achieve long-lasting benefit.^1^

 In the present study, ACS provided significant reduction in pain at 3 months and this effect was sustained up to 1 year. The reduction in pain scores was also associated with a significant improvement in the functional scores. Considering the limited efficacy of both glucocorticoids and platelet-rich plasma in pain relief, intratendinous injection of ACS is likely to be more effective than corticosteroids or PRP in patients with LE.

 Orthokine^®^ differs from platelet-rich plasma and autologous whole blood by being prepared using a method that allows activation of monocytes. The resulting preparation has been demonstrated to be rich in IL-1ra, IL-4, and IL-10.^17^ An *in vitro* study reported that intra-articular injection of ACS increased the level of anti-inflammatory cytokines such as IL-1ra, transforming growth factor beta, and IL-10, as well as increasing the levels of the pro-inflammatory cytokines IL-1beta, IL-6, tumor necrosis factor alpha, and oncostatin M. However, less is known about the effects of ACS on tendon healing, as most of the studies focused on cartilaginous diseases.^8^

 One randomized study compared ACS injection to steroid injection in LE patients with regard to the Disabilities of the Arm, Shoulder and Hand (DASH) scores at 6 weeks, 6 months, and 1 year after treatment.^18^ At 6 weeks and 6 months, ACS injection was not better than steroid injection, while the DASH scores of the ACS group improved at 1 year, indicating that steroids may result in rapid improvement, but ACS provides longer-lasting effects. Although the manufacturer’s technique for ACS preparation was different from the technique we used, the outcomes at 1 year were like ours.

 In contrast, another randomized pilot study enrolling 28 patients compared ACS + dry needling and dry needling alone in patients with refractory LE. The authors noted no significant difference between the two groups at 2-month or 6-month of follow-up.^19^ However, the study was limited by its small sample size, and the patients who received ACS tended to have better pain and better Nirschl scores. Like our results, the study shows the potential of ACS to provide pain relief in both short- and long-term.

 Schöffl et al recently evaluated the efficacy of ACS in treatment of LE in a placebo-controlled study.^11^ The study randomized a total of 50 patients into an ACS injection group (performed a total of 3 times) and a placebo group, and follow-up was performed using the VAS pain score and DASH score at 4 weeks and 6 months. The authors reported that there were no significant differences in outcome measures between the two groups at 4 weeks and 6 months. Interestingly, the decrease in DASH scores at the two follow-up points was significant in the placebo group but not in the ACS group, and the authors hypothesized that the improvement seen after injection therapy in both groups was due to the local anesthetic, not the preparation itself. Given that the local anesthetic was given only before the first application in both groups, and 14 of the 50 patients were lost to follow-up, this hypothesis awaits confirmation.^11^

 The main limitations of the present study were its small sample size and non-comparative design, which preclude us from reaching a definitive conclusion on the use of intratendinous injection ACS for the treatment of chronic LE. Although perceived as a comfortable method, it is an invasive procedure as it requires obtaining blood from the patient. Moreover, treatment may not be applicable to all patients since the cost of this procedure is high and it is not reimbursable. Based on our findings, further larger scale prospective studies are needed to confirm the efficacy and safety of Orthokine^®^ in treatment of LE in an outpatient setting.

 In conclusion, intratendinous injection of ACS is a promising option for short-term pain relief of LE symptoms and to achieve long-lasting functional improvement in patients with LE. Our results await confirmation by large-scale and well-designed prospective trials.
